# P-476. Pilot Implementation of HIV Self-Testing in Ghana: Analysis of the Community-Based Demand Generation Approach

**DOI:** 10.1093/ofid/ofae631.675

**Published:** 2025-01-29

**Authors:** Ernest Amoabeng Ortsin, Jonathan Tetteh-Kwao Teye, Richard Socrate Adzesi, Kwadwo Owusu, Anthony Ashinyo, Stephen Ayisi Addo

**Affiliations:** Ghana HIV and AIDS Network (GHANET), Accra, Greater Accra, Ghana; Dreamweaver Organization, Accra, Greater Accra, Ghana; Ghana National TB Voice Network, Accra, Greater Accra, Ghana; National AIDS/STI Control Programme, Accra, Greater Accra, Ghana; National AIDS/STI Control Programme, Accra, Greater Accra, Ghana; National AIDS/STI Control Programme, Accra, Greater Accra, Ghana

## Abstract

**Background:**

Under the auspices of the Ministry of Health (MoH) and National AIDS/STI Control Programme (NACP), Ghana piloted an HIV Self-Testing (HIVST) project in 2023. The implementation took place in 50 HIV high burden districts and it was spearheaded by Ghana HIV and AIDS Network (GHANET), a grassroots civil society organization (CSO) with membership across the country. This paper examines the strategies and outcomes of the project which was sponsored by the Global Fund and currently being expanded as part of Grant Cycle Seven (GC7) implementation from 2024 to 2026.

**Methods:**

One hundred and fifty (150) volunteers from 50 community-based organizations (CBOs) were trained to distribute ORAQUICK HIVST kits to local residents in the selected districts. Video instructions on the effective use of the test kits (produced in English and six different Ghanaian dialects) were made available to persons who voluntarily received the kits. Through an online portal (GHANET Information Management System (GIMS)) data about the distributions were recorded. Follow-ups were conducted to ascertain, among others, how the kits were used. The data was quantitatively analyzed and descriptive statistics was used to summarize the data using Statistical Package for Social Science (SPSS v.28).

**Results:**

A total of 123,088 ORAQUICK HIVST kits were distributed (with follow-ups) to 73,853 (60%) females and 49,235 (40%) males. Young people aged 20 – 24 received the highest number of kits totaling 28,790 which is about 23%, followed by those aged 25 – 29 who received 25,006 which is about 20% and those aged 30 – 34 who received 17,460 which is about 14%. A total of 422 persons comprising 304 (72%) females and 118 (28%) males self-reported their results as reactive. Of this number, 329 persons comprising 72% of females and 28% of males were confirmed positive. And, out of that number, 202 persons comprising 73% females and 27% males were successfully initiated on antiretroviral therapy (ART) in health facilities.

**Conclusion:**

Male participation of 40% in the testing exercise was encouraging as it above the national average of 20% . It is therefore recommended that in Ghana, HIVST should, particularly, be targeted men.

**Disclosures:**

**All Authors**: No reported disclosures

